# Processing of Layered Composite Products Manufactured on the Basis of Bioresin Reinforced with Flax Fabric Using Milling Technology

**DOI:** 10.3390/ma17184528

**Published:** 2024-09-14

**Authors:** Wiesław Frącz, Grażyna Ryzińska, Leszek Skoczylas, Łukasz Bąk, Grzegorz Janowski

**Affiliations:** 1 Department of Materials Forming and Processing, Rzeszow University of Technology, Powstancow Warszawy 8, 35-959 Rzeszow, Poland; lbak@prz.edu.pl (Ł.B.); gjan@prz.edu.pl (G.J.); 2 Department of Manufacturing Processes and Production Engineering, Rzeszow University of Technology, Powstancow Warszawy 8, 35-959 Rzeszow, Poland; lsktmiop@prz.edu.pl

**Keywords:** layered composites, laminates, roughness parameters, bioresins, milling of layered composites

## Abstract

In this work, a laminate based on bioresin and natural fibers was produced. Flax fabric was selected as the natural fiber. The biocomposite was subjected to strength tests. Stress–strain characteristics and strength indicators were determined. The workability of the laminate produced was also tested using milling technology. The tests were carried out using five carbide shank cutters for different purposes. The cutters with the geometry used in the processing of polymer materials and composites, general purpose cutters, and cutters with the geometry for aluminum and with different numbers of blades were analyzed. In order to obtain information on the workability of the prepared material, machining tests with different configurations of technological parameters were carried out. For each cutter, the effect of cutting speed and feed rate on the quality of the machined surface was tested. Due to the small thickness of the laminate, the machining was carried out in one pass, as a result of which the cutting depth in each case was constant. Changes in cutting speed and feed were evenly distributed over five levels. The quality of machining was assessed in two stages. The first stage included a visual assessment of the machined surface, involving a preliminary qualification of the machining parameters. The criterion was the amount of chips, frays, burrs, etc., remaining after machining that adhered to the surface. The next stage was the measurement of the geometric structure of the surface, during which the roughness parameters were analyzed using an optical microscope with a roughness analysis attachment. Quantitative analysis was performed for the best quality composite surfaces from each measurement series. The studies showed a dependence of the quality of machining on the technological parameters used. High tool speed, regardless of the type, especially at low feed, led to the sticking of chips, which had a very delicate form. In turn, low tool speed and high feed, due to the chip thickness, favored the formation of burrs. Machining with different types of tools showed that the process progresses better for tools with sharp blade geometry. Machining with a regular and polished cutter did not show any differences in the scope of the process progress.

## 1. Introduction

The increasing amount of plastic waste originating from the petrochemical industry, often mixed with glass, carbon, or aramid fibres, the production of which is energy-intensive and contributes to greenhouse gas emissions, forces the search for new solutions. The answer is composite materials made of natural fibres (NFCs), easily available and biodegradable, which, when combined with a properly selected matrix, could constitute a product with excellent utility properties for various applications.

In recent years, natural fiber composites have become an increasingly common and sought-after alternative to traditional combinations of carbon, glass, or aramid fibers with thermosetting or chemically cured resins or thermoplastics, constituting a modern material for engineering applications [1,2,3,4]. However, the most important thing is to develop composites of this type from the point of view of minimal impact on the natural environment in the context of the greatest possible reduction of greenhouse gas emissions.

Natural materials as composite reinforcement are gaining popularity due to their low price, non-toxicity, easy recycling, and, above all, a favorable ratio of mechanical properties to density. In addition, natural fibers significantly improve the acoustic insulation of the product. Natural fabric absorbs vibrations twice as well as glass fabric, and also improves thermal insulation by 15–30% compared to glass fabrics [5,6,7,8]. As a result, the global market for natural fibers has been constantly growing in recent years, and interest in these materials is gaining importance [9]. Natural fibers can come from plants, animals, and minerals. The group of plant materials that have attracted the attention of researchers in the context of the possibility of using them as reinforcement in composite materials includes products derived from plant bast (flax, jute, ramie, hemp, kenaf), leaves (agave, sisal, banana leaves), seeds (cotton, kapok), fruits (oil palm), wood (hardwood, softwood), stems (corn, rice, wheat, oats, rye, barley), and grasses and reeds (bamboo, canary, esparto) [10,11,12,13].

The problem in using natural fibers as a reinforcement of composites is their low wettability, which affects the quality of the fiber–matrix connection and thus reduces the mechanical properties of the finished composite and promotes the penetration of moisture into the structure of this material. Therefore, a very important aspect from the point of view of using raw materials of natural origin as a reinforcement of the composite is to indicate the optimal methodology for preparing the reinforcement material so as to eliminate the problem of low wettability [14,15].

Flax fiber is one of the most popular and available fibers in Europe, where 80% of the world’s annual production of these fibers occurs. It is characterized by mechanical properties that can be compared to glass fibers, taking into account the ratio of their stiffness to density, which is almost two times lower than that of glass fibers [16,17]. Flax fibers are cheap, lightweight, and have excellent vibration-damping properties. Moreover, their production has a small impact on the natural environment. The energy demand necessary for the production of flax fibers is about 10 MJ/kg, while the production of glass fibers uses about 50 MJ/kg. Therefore, flax is one of the most commonly used biofibers. 

The use of flax fibres as reinforcement for composite materials is becoming more and more widespread and includes, for example, elements of snowboards, tennis rackets, window frames, bicycle frames, terraces, and fences [8].

Combining natural fibres with synthetic matrices is a half-way solution from an ecological point of view; hence, in recent years, biodegradable or biologically derived materials have been sought that can act as matrices in composites, which will allow for a gradual reduction or elimination of petrochemical industry products. Such a solution may be bioresins, which are partially or completely natural in origin, thus reducing greenhouse gas emissions and maintaining high-utility properties [18,19,20]. Consumers expect a gradual introduction of products based on renewable resources, which are generally available, cheap, and non-toxic. These properties are increasingly used in the research and development of obtaining epoxy bioresins. A very important application of this type of resin may be matrices in composites reinforced with natural fibers, which will allow obtaining construction materials that are safe for the environment, competitive in terms of production costs, and comparable or even better in terms of selected properties in engineering applications. The problem is to develop and appropriately adjust the finishing conditions for this type of material, which differ significantly from typical composites, and in particular the selection of tools and process parameters that will allow for obtaining the best possible surface quality of the finished product.

Composite materials, due to their properties, constitute an important group among construction materials. In the conditions of developing new composites, we rarely have appropriate specialized devices for preparing samples for testing (e.g., water cutting devices). In such cases, it is easiest to use standard methods. An analysis of the literature indicates that machining is the most commonly used technology in industry for preparing samples and semi-finished products in small series from composites. Problems in machining composites result from different properties of the material components. While the matrix is relatively well-machinable (resins, light metals, polymers), composite reinforcements may be characterized by different machinability. Hence, the main problems occurring in composite machining are delamination, fraying, burrs on the machined surface, high roughness, and a change in composite properties resulting from the high temperature accompanying machining. Problems also concern tools, which, depending on the composite, may become blunt or wear out quickly. Hence, the appropriate selection of tools, their geometry, the material they are made of, and the processing parameters have a decisive influence on the obtained effect and in many cases must be preceded by a series of tests. The selection of machining technology and parameters determines the quality of the obtained machining products. This is confirmed by publications in the conducted analysis of the literature on the machining of composites with continuous plant fibers.

In drilling operations, for example, it is mainly about obtaining the appropriate hole quality. In the publication [21], the authors studied the drilling process in composites reinforced with natural flax fibers. It was crucial to investigate how the feed, spindle speed, and drill type affect the axial force, delamination size, and surface roughness of drilled holes. The research was carried out on composite laminates made of satin flax weave, reinforced with epoxy resin. The research showed that the type of drill and feed have a key impact on drilling quality. The twist drill was the most effective in minimizing the axial force, delamination, and surface roughness. In the work [22], the authors focused on examining the effect of drilling parameters on minimizing delamination damage. The research showed that there is a critical feed value below which delamination damage is minimized. The model they developed allows for predicting the critical feed and minimizing damage. In turn, the research from publication [23] was focused on the study of damages caused during the drilling of flax composites with epoxy resin. The work showed that the proper optimization of drilling parameters can significantly reduce material damage. The influence of drilling parameters on the strength properties of flax–epoxy composites with holes was also studied [24]. The research showed that the tensile strength of flax–epoxy composites with holes was mainly dependent on the configuration of the fiber arrangement. Unidirectional laminates had the highest strength, and bidirectional laminates the lowest. Drilling parameters had a smaller effect on strength, but were important in the context of delamination damage. The use of a step drill contributed to the reduction of delamination, which is beneficial for maintaining the mechanical strength of the composites. The work [25] presents the results for the residual tensile strength and delamination factor of drilled flax composites. Drilling parameters such as rotational speed and feed were shown to have a significant effect on residual strength and delamination, with the lowest damage achieved at the lowest feed rate and the highest rotational speed. Tool geometry had a smaller effect on the results. Similarly, in [26] it was shown that feed has the greatest effect on residual tensile strength. Increasing feed rate causes higher axial forces, which in turn leads to greater damage and a decrease in tensile strength. Spindle speed has a moderate effect on residual strength, while drill geometry has the smallest effect. Similarly, in milling operations, research focuses on obtaining the best possible surface quality. In [27], the cutting properties of composites reinforced with jute fibers (JFRPs) and flax fibers (FFRPs) were analyzed. The authors focused on determining the optimal parameters to minimize delamination damage and maximize surface quality after machining. High-speed steel (HSS), TiN-coated, and tungsten carbide (WC) milling cutters were used in the study. It was noted that FFRP composites had a higher delamination rate than JFRP. The lowest delamination rate values were obtained using WC cutters, and the highest using HSS cutters. Higher spindle speeds and feeds increased the delamination rate. Surface roughness was higher for FFRP composites than JFRP. The lowest roughness values were obtained using WC cutters, and the highest using HSS cutters. Higher spindle speeds decreased roughness, while higher feeds increased it.

In [28], an attempt was made to machine epoxy resin composites reinforced with unidirectional and bidirectional flax fiber fabric. Two cutting tools were used for the tests: a CVD diamond-coated six-flute cutter and a two-flute cutter with PCD diamond blades. The tool with PCD blades provided better results in terms of cutting forces, surface finish, and fewer uncut fibers compared to the CVD tool. The best results were obtained for fibers oriented in the cutting direction (0°), while the worst surface finish was observed for fibers oriented at an angle of ±45°. In drilling operations, the main goal is to obtain the appropriate hole quality. The authors of the publications [29,30,31] mainly investigated the effect of cutting parameters (cutting speed, feed, drill type) on the size of delamination and the surface roughness of drilled holes, axial force, and strength properties of flax–epoxy composites with holes.

The machining of composites made of thermosetting resins reinforced with plant fibers, such as flax fibers, requires special attention in the selection of tools and optimization of cutting parameters. The selection of machining technology and parameters determines the quality of the obtained products and the accuracy of determining material properties. It turns out that these parameters must be selected each time, adequately to the materials used in the composite, i.e., matrix and reinforcement. Work carried out in this area confirms problems with determining the best processing parameters.

A very important aspect of using the new composite is the finishing of products, and in particular the cutting method, which allows for obtaining very good surface quality. For this purpose, this research proposed the development and production of a composite material based on bioepoxy resin of 60% natural origin and natural flax fabric, as well as the selection of cutting tools and optimal parameters for this process. Using the example of the composite material produced, milling machining tests were carried out using tools of different geometry with different processing parameters, with the aim of obtaining the best possible surface quality. Due to its high visual appeal, the obtained composite material, reinforced with flax fabric based on bioepoxy resin, can play a decorative role. Ultimately, the considered biolaminate can be used, among others, in the production of boxes, packaging, containers, casings, panels, and screens.

## 2. Materials—Manufacturing

**Figure 1:**
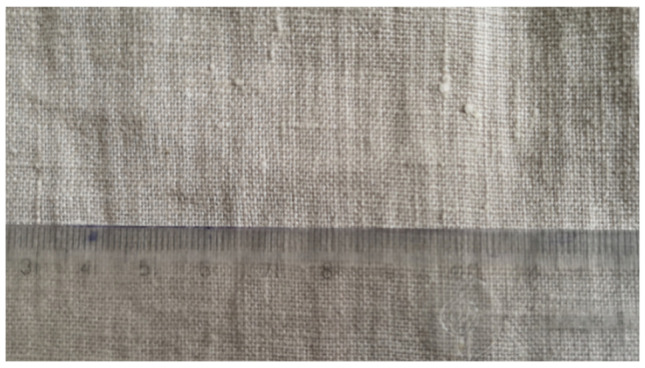
Flax fabric with a plain weave and a weight of 200 g/m^2^ used in the tests.

**Figure 2:**
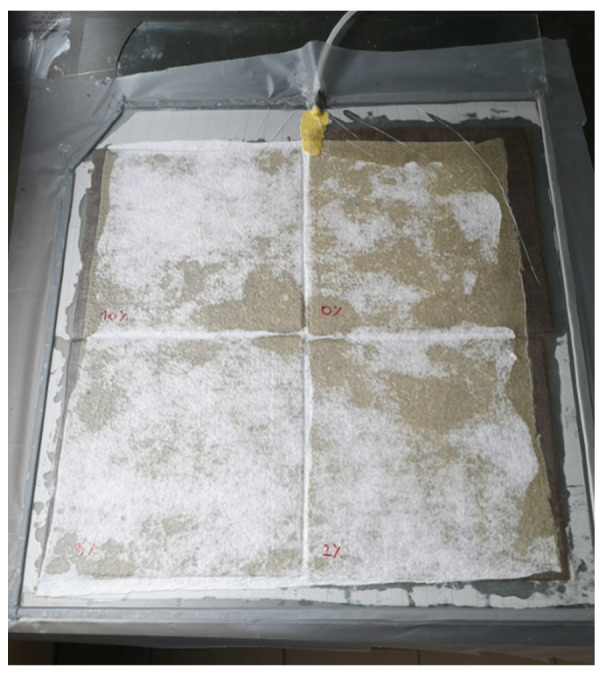
Composite panels in a vacuum bag.

To produce the composite, a flax fabric with a plain weave and a density of 200 g/m^2^ was used (Figure 1). A portion of resin and slow hardener was measured and then mixed in a container (mix ratio 3R:1H by volume). The mixture was left for a specified time to eliminate air bubbles. The fabric was cut to the appropriate size and manually permeated with Ampro Bio resin. Four composite boards with dimensions of 450 × 450 mm were made (Figure 2). Six layers of fabric were used. Then, all boards were closed in a vacuum bag and the air was sucked out. The curing process was carried out at room temperature.

## 3. Mechanical Properties of the Composite

The tests of the mechanical properties of the biocomposite were carried out on a Zwick/Roell Z030 testing machine (Ulm, Germany). The results are presented in the table (Table 1). For comparison, the properties of the Ampro Bio resin specified by the manufacturer (Gurit, Wattwil, Switzerland) are presented (Table 2).

**Figure 3:**
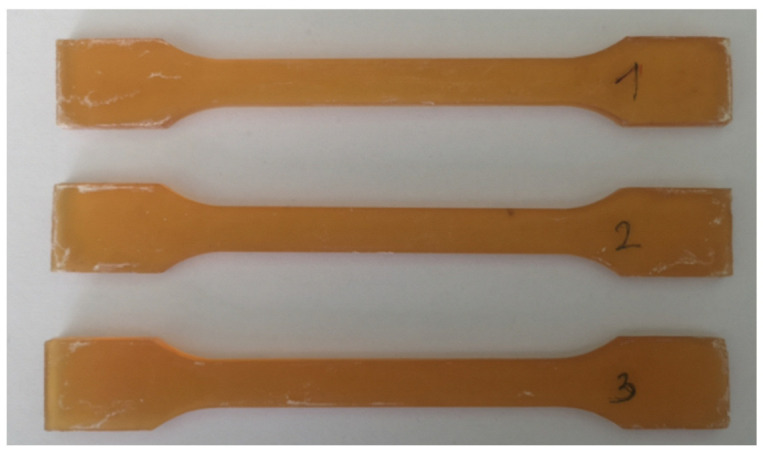
Examples of bioepoxy resin samples used in the tests.

**Figure 4:**
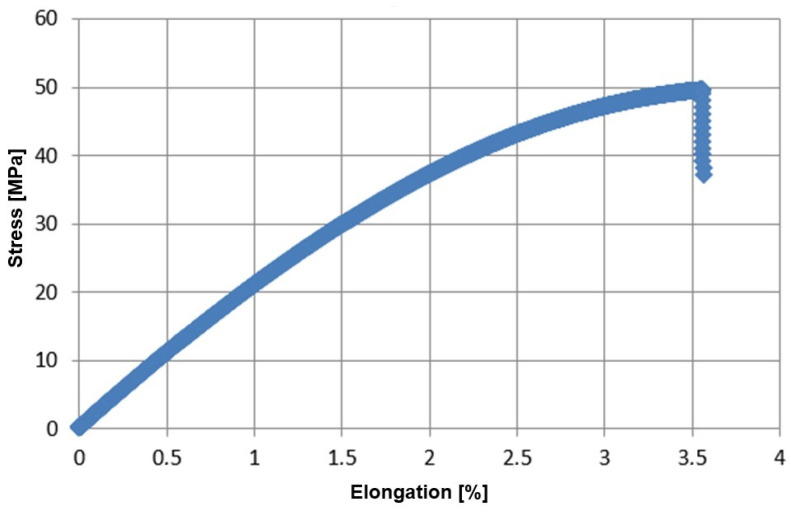
Stress–strain relationship for AmproBio resin.

The typical stress–strain relationship for the tested bioepoxy resin specimens (Figure 3) is presented in Figure 4.

**Figure 5:**
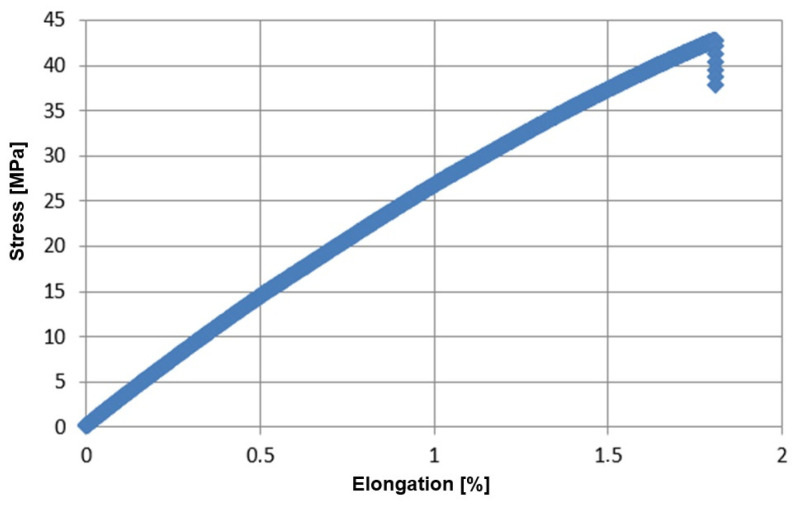
Stress–strain relationship for typical LH288 (HAVEL) epoxy resin.

For comparison, the characteristics of a typical, classic epoxy resin are presented (Figure 5), for which standard mechanical properties were determined (E_t_ = 2921.23 MPa, σ_x1_ = 29.02, σ_M_ = 43.47 MPa).

**Figure 6:**
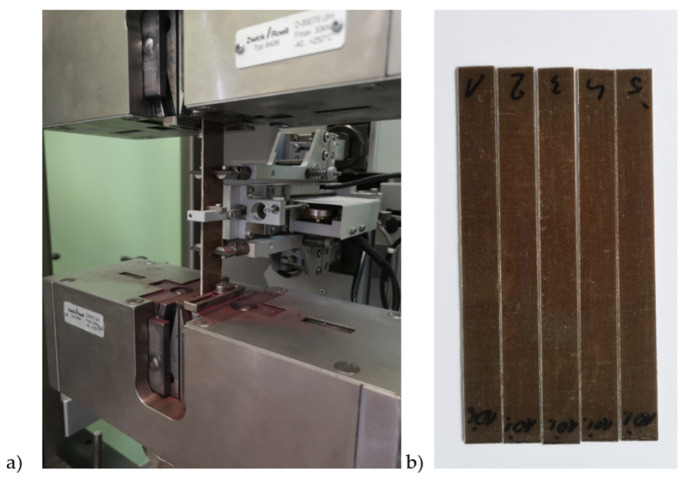
Workspace of the stand for testing the mechanical properties of the material in a uniaxial tensile test (**a**), samples for carrying out a static tensile test (**b**).

**Figure 7:**
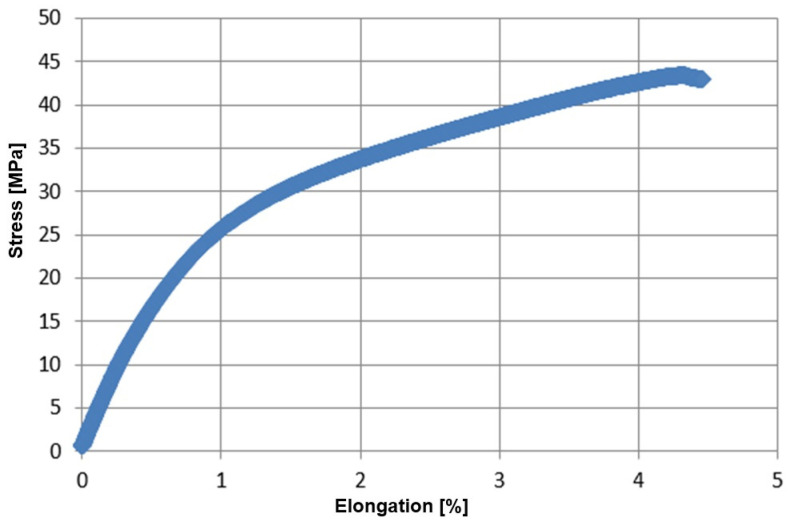
Example of stress-elongation relationship for the tested flax fabric–bioepoxy resin composite.

Samples made of the tested composite were subjected to a uniaxial tensile test on a Zwick/Roell testing machine (Figure 6). The results are given in Table 3. A typical stress–strain characteristic is shown in Figure 7. The test stand for the tensile test and sample composite samples are shown in Figure 6.

## 4. Composite Machining Using the Milling Process

**Figure 8:**
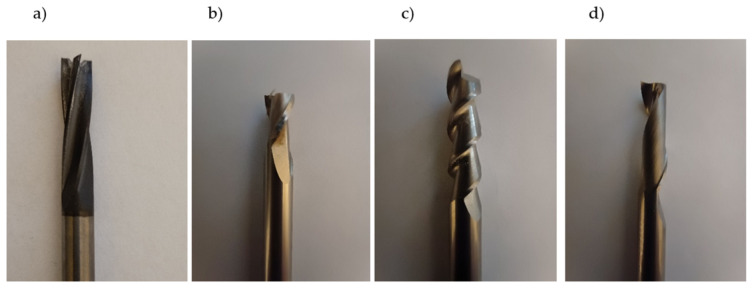
Photographs of the milling cutters used in the tests: (**a**) four-flute milling cutter for composites SCM 400, (**b**) universal milling cutter for steel, (**c**) two-flute carbide milling cutter for aluminum, (**d**) single-flute carbide milling cutter for aluminum.

Taking into account the research aims of this study, shank cutters of various purposes and a wide range of changes in processing parameters were used for the tests. The tests were conducted on a table milling machine with a spindle with a maximum rotational speed of 18,000 rpm. A photograph of the tools used is shown in Figure 8. The first tool is a Mapal SCM400 series cutter (Figure 8a). It is a four-flute carbide cutter intended for composite processing. The next cutters are Dolfamex tools. The first one (Figure 8b) is a universal cutter designed for steel processing, a two-flute carbide cutter made in accordance with the DIN 6527 AN standard [39]. The next cutter (Figure 8c) is a two-flute carbide cutter for aluminum (Dolfa2-Al). The remaining cutters (Figure 8d) are also carbide cutters for aluminum, only single-flute. Due to the fact that the single-edge milling cutter is available in a regular version (Dolfa1-Al) and a polished version (Dolfa1S-Al) with the same blade geometry, both variants were used for the tests. All the milling cutters used have a diameter of 6 mm.

**Figure 9:**
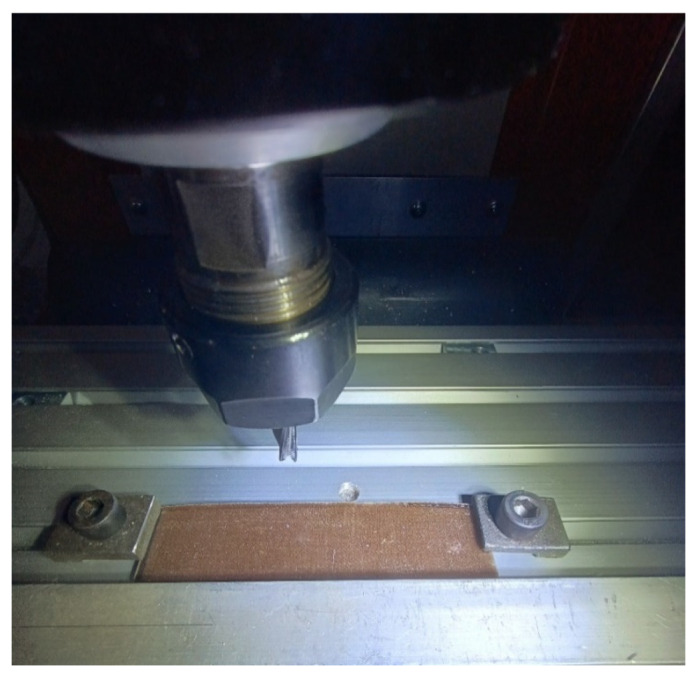
Sample fixing during milling.

In milling operations, the main parameters influencing the machining result are: cutting speed, feed speed, and cutting depth. In the case of cutting or cutting out appropriate shapes in thin plate materials, the process is carried out in one pass of the tool. Such a processing course means that the cutting depth remains an unchanging parameter and the remaining two parameters are subject to changes. Due to the small thickness of the processed material, the processing was carried out on the full thickness of the material in one pass (Figure 9). Subsequent passes were performed parallel from the front of the plate at a length of 15 mm. It was assumed that such a processing course would provide repeatable conditions in terms of the stiffness of the processed plate for each pass of the tool. Taking into account the fact of the cross arrangement of the reinforcing fibers of the composite, the tests were carried out at an angle of 0 and 45 degrees to the fiber direction. This will allow determining the effect of the tool movement direction on the quality of processing.

**Figure 10:**
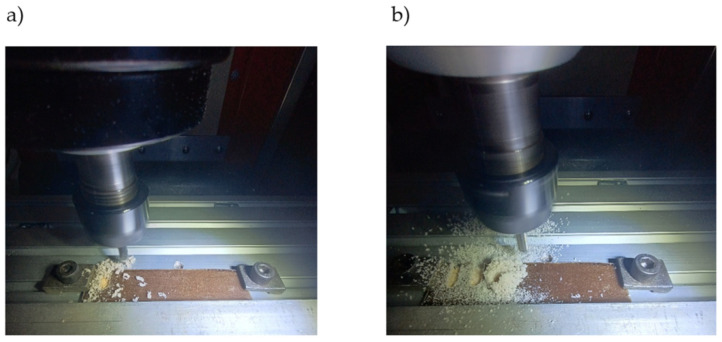
Consequences and effects occurring during the milling of biocomposite boards: for a low spindle speed and a relatively high feed (**a**), for high spindle speed and a low feed speed (**b**).

According to the manufacturer’s recommendation (Dolfamex, Jelenia Góra, Poland), the cutting speed for carbide cutters for machining reinforced plastics is 90 ÷ 160 m/min. The recommended feed per tooth for this type of machining is 0.045 mm/tooth. Due to the lack of information on the cutting conditions of the composite that is the subject of this study, preliminary milling tests were carried out. An example of the shape of chips and the implementation of subsequent tool passes are shown in Figure 10. The first case is characteristic for a low spindle speed (Figure 10a) and a relatively high feed. In turn, the second case corresponds to a high spindle speed and a low feed speed (Figure 10b).

**Figure 11:**
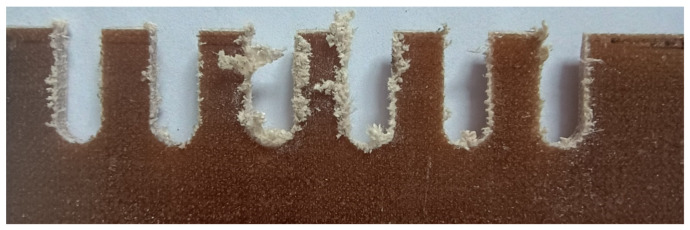
Example of the cutting effect of a selected composite sample with different technological parameters—view from the front surface.

For this type of processing, depending on the parameters, different effects are obtained. As shown in Figure 11, subsequent passes differ significantly from each other.

The lack of information on the machinability of the tested material led to the adoption of a large area of changes in the tool rotational speed and its feed with several intermediate levels. This will allow for the observation of changes in the machining effects for the variant of constant tool rotational speed and constant feed rate. Based on the preliminary tests and observations, it was assumed that the tool rotational speed would change in the range of 1000–9000 rpm, and the feed would be in the range of 100 ÷ 900 mm/min. For such an assumed range of changes in the rotational speed, the cutting speed for tools with a diameter of 6 mm is 19–170 m/min, respectively. Converting the feed speed to the feed per tooth for the assumed range of changes, we obtain: 0.011 ÷ 0.9 mm/tooth for a single-tooth cutter, 0.005 ÷ 0.45 mm/tooth for a double-tooth cutter, and 0.0025 ÷ 0.225 for a four-tooth cutter.

In order to determine the nature of changes in the influence of rotational speed and tool feed on the machining effect (measured parameter characterizing surface roughness or its jaggedness, burrs, uncut fibers), it was decided to carry out cutting tests of the produced composite based on the plan presented in Table 1.

The adopted levels of parameter variation correspond to the implementation of the complete three-level static plan. Tests according to the complete three-level plan may refer to the entire research area and then the tool rotational speed levels will be 1000–5000–9000 rpm, and the feed 100–500–900 mm/min. They may be narrowed down to a smaller range and then the tool rotational speed areas (1000–3000–5000 rpm, 3000–5000–7000 rpm, 5000–7000–9000 rpm) and feed (100–300–500 mm/min, 300–500–700 m/min, 500–7000–9000 rpm) may be checked. The complete three-level plan allows for the development of a second-degree polynomial, which is associated with the possibility of the occurrence of an extremum and, in the case of the adequacy of the model, the possibility of determining the optimal machining parameters with respect to the adopted criterion.

## 5. Surface Quality Tests after the Milling Process and Results Analysis

**Figure 12:**
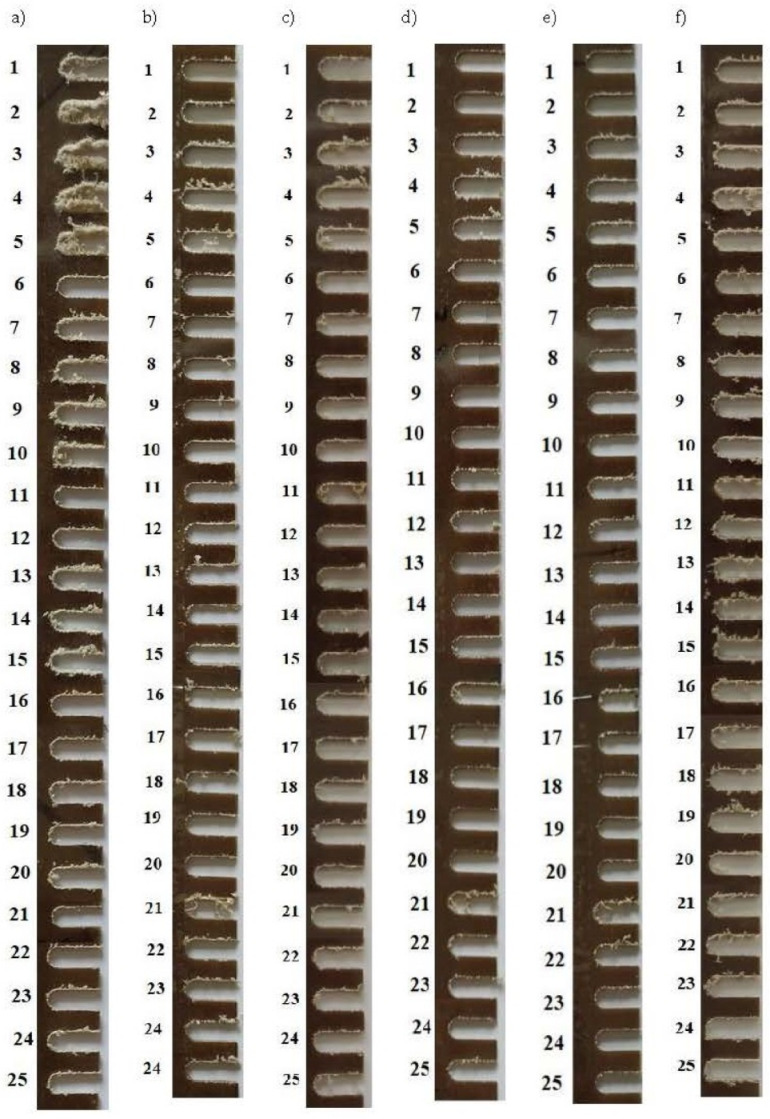
Photographs of the laminate processed in accordance with the adopted plan (Table 4) using the following tools: (**a**) composite cutter, (**b**) steel cutter, (**c**) two-flute cutter for aluminum, (**d**) single-flute cutter for aluminum, (**e**) polished single-flute cutter for aluminum, (**f**) two-flute cutter for aluminum-machining at an angle of 45°.

The high variability of machining parameters and different tool geometries resulted in significant differences in the quality of the machined surfaces for the tested samples. Therefore, the quality of machining was assessed in two stages. The first stage included a visual assessment of the machined surface, involving a preliminary qualification of the machining parameters. The criterion was the amount of chips, frays, burrs, etc., remaining after machining and adhering to the sample surface. The next stage was the measurement of the geometric structure of the surface, where the roughness parameters were analyzed using an optical microscope with a roughness analysis attachment. In accordance with the assumed research plan, 25 milling tests were carried out for each tool (five levels of cutting speed at five levels of feed speed). For the tools used in the tests, 125 samples were made, only for the direction of tool movement parallel to the direction of reinforcement fibers. Additionally, for the two-edge milling cutter for aluminum, machining was performed at an angle of 45° in relation to the fiber arrangement. Photographs of the milled samples are shown in Figure 12.

Visually analyzing the machined surfaces of the composite cutter samples (Figure 12a), significant differences in their quality can be seen at different levels of machining parameters. As the tool rotational speed increases, the amount of residue stuck to the chip surface increases. Increasing the feed rate improves the machining effects, but does not eliminate the problems completely. It should be noted that this is a four-flute cutter, so the chip thickness is the smallest of all the tools for the given levels of machining parameters. Assessing the subsequent passes, it can be seen that the least residues occur at high feed rates (700–900 mm/min) and low tool rotational speeds (1000–3000 rpm). The worst machining quality occurs for low feed rates (100 mm/min) and rotational speeds above 3000 rpm.

Considering the machining performed with a milling cutter designed for steel machining (Figure 12b), a similar phenomenon can be observed. An increase in the rotational speed promotes a greater amount of material residue removed from the machined surface, but not with such intensity. An increase in the feed speed also improves the situation, but does not eliminate it. In the range of feed parameters 300–700 mm/min, no significant changes in the amount of material residue removed from the surface of the machined item are observed. The worst situation is at a low feed (100 mm/min) and high tool rotational speed (7000–9000 rpm) and high feed (900 mm/min) in the entire speed range.

A two-flute milling cutter with geometry for aluminum is another tool used in machining (Figure 12c). Also in this case, the increase in the tool rotational speed at a low feed promotes the adhesion of chips to the laminate surface. The worst is the case with a feed of 100 mm/min and a rotational speed of 5000–9000 rpm. The feed range of 300–700 mm/min allows for a similar effect with a slight deterioration of the surface quality after machining for higher tool rotational speeds.

Single-flute milling cutters for aluminum were also analyzed (Figure 12d,f). Machining with these milling cutters was characterized by the highest thickness of removed chips. Differences in machining with a regular and polished milling cutter at the stage of visual assessment are imperceptible. It should be noted, however, that these milling cutters, among the milling cutters tested, provided the best machining effect in terms of visually assessed surface quality of the machined material. A relatively clean surface was obtained at low feed parameters (100) and tool revolutions (1000–3000). Good quality was also obtained at higher feeds but also high tool rotation speeds. High feed (700–900 mm/min) and low tool rotation speed (1000–3000 rpm), due to the large thickness of chips, caused the opposite effect, which resulted in an increase in the amount of residue.

Figure 12f shows the results of machining the composite with a two-edge milling cutter for aluminum with the tool moving at an angle of 45° to the reinforcing fiber line. A comparison with machining parallel to the fibers (Figure 12c) shows a deterioration in the quality of machining in the entire range. Thus, the direction of the milling cutter movement in relation to the fiber alignment affects the quality of machining.

When characterizing the machining, attention should also be paid to the stability of its course. In the case of cutters with a large flute twist at a high feed and low rotational speed, which corresponds to a large chip thickness, the axial force of the tool pulls the processed material away from the machine table, resulting in delamination and burr formation. In addition to the deterioration of the surface quality, it adversely affects the course of the milling process. This phenomenon was most visible for the two-flute milling cutter for aluminum. The milling cutter for composites, due to the smallest flute twist, caused the smallest pull of the laminate away from the table surface. Comparing the machining with subsequent tools, it can be seen that, according to a visual assessment, the best effect was obtained for single-flute milling cutters for aluminum, while the worst was for the four-flute milling cutter for composites.

**Figure 13:**
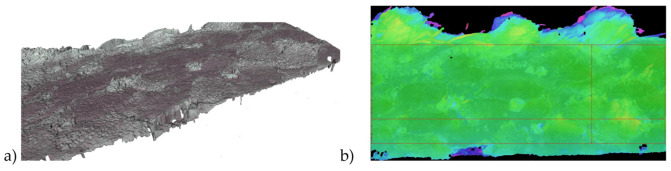
Side surface of the composite: (**a**) surface scan, (**b**) analyzed area of the sample.

The analysis presented above refers to the residues of chips, frays, and burrs that stick to the surface after machining. These results do not indicate the quality of the side surface, directly shaped by the tool. To determine the quality of the side surface after milling, measurements of the geometric structure of the surface were taken. Roughness measurements were performed on an Alicona Infinite Focus G4 microscope. An example scan of the machined surface and the analyzed area are shown in Figure 13.

Roughness tests of the machined side surface were performed only for selected samples. This was due to the fact that the large fraying of the machined surface prevented optical scanning of the side surface of the sample, and thus roughness measurement. Therefore, the best machined samples according to visual assessment were selected for measurements (Figure 12). Roughness parameters were determined for the scanned laminate surfaces using software. The samples selected after the initial assessment for roughness assessment, together with the calculated parameters, are presented in Table 5.

**Figure 14:**
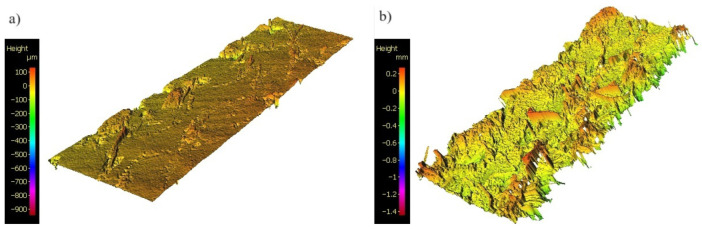
Comparison of surface topography for the parameters of spindle speed of 3000 rpm and feed of 700 mm/min for milling cutters: (**a**) single-flute polished for aluminum; (**b**) four-flute for composites.

The average height of area (Sa) roughness parameter was used to assess the roughness of the side surface after milling. Comparing the measurement results, it can be seen that the highest values are observed for the composite tool (Mapal SCM400), where the values reach values of over 20 µm. It should be noted that this applies to the best-processed samples. All scanned side surfaces show frayed fibers and recesses from pulled-out fibers (Figure 14), which affect the value of the Sa parameter. The roughness analysis confirms the observations of the visual analysis, where the largest amounts of material removal residues can be found on the surface for this tool (Figure 12 and Figure 14).

**Figure 15:**
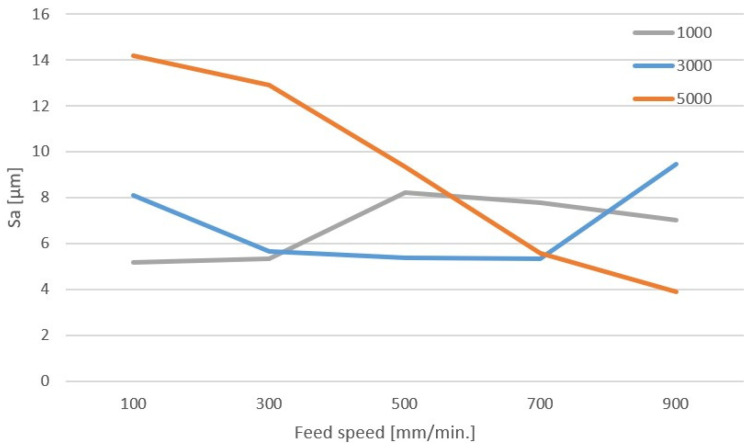
Characteristics of roughness changes for machining with a double-edge tool for aluminium.

For the remaining tools, the roughness of the machined surface is in the range of 3–14 µm. To better illustrate the changes in roughness parameters for aluminum milling cutters, characteristics of changes in the Sa parameter were made depending on the feed and rotational speed of the tool. The graph of changes for the two-flute milling cutter is shown in Figure 15. This figure takes into account three levels of tool rotational speed (1000, 3000, 5000 rpm) in the entire feed range. The remaining speed levels were omitted because an increase in the amount of chip residue on the surface was observed for the highest speeds.

**Figure 16:**
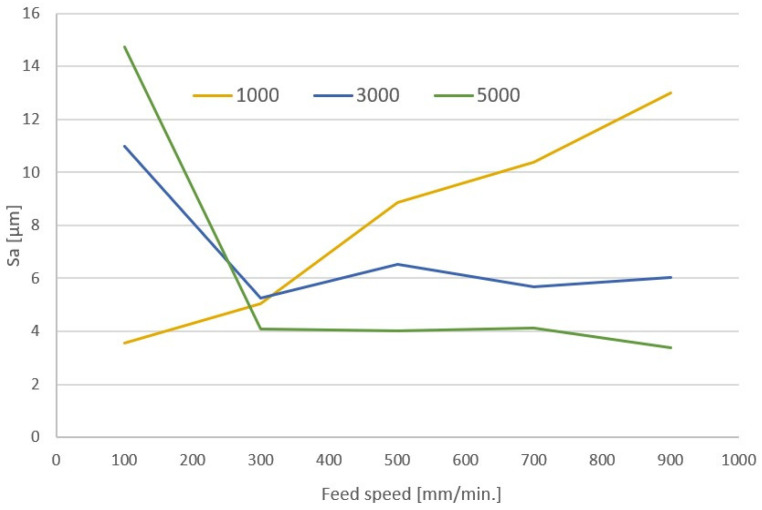
Characteristics of roughness changes for machining with a single-edge polished tool for aluminum.

When analyzing the characteristics, a certain regularity can be noticed that was noticeable during visual assessment—high rotational speed and low feed led to large amounts of chip residue and a high value of the Sa parameter in roughness measurements. For a speed of 3000 rpm, a course with a local minimum is observed around the feed of 500 mm/min. Comparable surface roughness for this rotational speed is in the feed range of 300–700 mm/min. The cutting speed for 3000 rpm is about 56 m/min, while the feed per tooth is 0.05–0.12 mm. Similar characteristics were prepared for machining with a single-flute cutter (Figure 16). The characteristics show a similar relationship as in the case of a double-flute cutter.

For a spindle speed of 1000 rpm, the lowest surface roughness was obtained for a feed of 100 mm/min. The high quality of machining for these parameters was also confirmed based on the assessment of the surface after machining (Figure 12e). With the increase in the feed speed, the value of the Sa parameter increases. In the case of spindle speeds of 3000 rpm and 5000 rpm for low feed speeds (100 mm/min), a high value of the Sa roughness parameter was obtained. Increasing the feed speed to 300 mm/min causes a rapid decrease in this parameter. Further increases in the feed speed do not cause significant changes in the Sa parameter values. Thus, the roughness analysis also confirms the observations of the visual analysis. With a small amount of residue, a lower roughness of the machined side surface of the laminate is observed. A minor deviation from this observation is the surface roughness for the tool movement at an angle of 45° in relation to the reinforcing fiber line. Despite a slightly larger amount of residual material removed from the machined surface, the roughness parameter Sa remains at a similar level as when the tool moves parallel to the grain line.

## 6. Conclusions

The introduction of a new construction material to the market is always associated with conducting a series of tests. They concern both the operational properties of the material, which determines its use, and the technological properties related to its shape. The strength of the presented material proves the possibilities of its use as a substitute for laminates used in the processes of manufacturing elements exposed to small loads, while ensuring the possibility of biodegradability after the period of use.

Referring to the machining issues of the tested material, it can be stated that a high rotational speed of the tool, regardless of its type, especially at low feed, leads to the sticking of chips, which have a very fine form. The probable cause of this phenomenon is the increased temperature resulting from the high rotational speed and the large angle of wrapping of the tool by the material. This can be confirmed by the characteristic smell during the machining process. In turn, a low rotational speed of the tool and high feed due to the thickness of the chip promote the formation of burrs. This method of machining introduces instability in its course. Comparing the machining with the tested types of tools, a better course of the process was found for tools with a sharp blade geometry (cutters for aluminum). The direction of the tool movement in relation to the arrangement of the reinforcing fibers of the composite also affects the quality of machining. Comparing the tools, the best machining effect was obtained using a single-flute milling cutter with geometry for aluminum. Machining with a regular and polished milling cutter did not show any differences in the scope of the process course.

Surface roughness tests showed that for selected samples based on visual assessment there is a correlation with machining parameters. Roughness measurement also confirmed the existence of differences between the tools used. Therefore, conducting research in relation to new materials is most justified and supported by good surface quality as well as the absence of chips and burrs on the surface, which require further cleaning work and generates costs. In this case, the conducted experiments indicate the areas of machining parameters for individual tools meeting these requirements.

In summary, our studies have shown that the tested material is characterized by lower strength compared to materials with similar purposes (from 30 to 50%—compared to the properties of reinforced composites with flax fabric on the basis of the literature (different resin, weave, grammage)). However, it should be noted that the use of epoxy resin based on natural ingredients promotes the biodegradability of the material, and thus may have a positive impact on reducing environmental pollution after the product’s service life. The composite can be used in the production of low-loaded structural elements (housings, covers). In addition, our studies have shown a high dependence of the quality of processing on the technological parameters used. For individual tools, they also indicated the ranges of parameters and directions of their changes that are conducive to obtaining good surface quality after processing. It was found that satisfactory surface quality cannot always be obtained by selecting technological parameters based on the similarity of parameters used for other composites. In the machining of new materials, it is often necessary to carry out a series of tests and studies in the field of determining effective processing parameters, and not only to follow the general recommendations of tool manufacturers.

## Figures and Tables

**Table 1:** Mechanical properties of Ampro Bio resin determined experimentally (according to ASTM D 638 standard) [32].

	E_t_	σ_x1_	σ_Y_	ε_Y_	σ_M_	ε_M_	σ_B_	ε_B_	ɳ
	MPa	MPa	MPa	%	MPa	%	MPa	%	
x	2192.92	22.99	46.81	3.94	47.45	3.53	36.57	4.51	0.389
s	40.0984	1.86031	-	-	1.94120	0.41866	19.5014	2.05441	0.03898
V	1.82853	8.09182	-	-	4.09028	11.8437	53.3191	45.5498	10.0123

**Table 2:** Properties of Ampro Bio resin according to the manufacturer [19].

Working Time Properties							
PROPERTY		UNITS	20 °C		**Test Method**		
Thin-Film Gel-time		h:min	01:54		-		
Pot-life (150 g, mixed in water)		h:min	00:43		Tecam Gel Time		
Tack-off Time		h:min	04:30		Internal Gurit Method		
Earliest Sanding Time		h:min	20:00		Internal Gurit Method		
Ambient Cure Thermal Performance Progression at 21 °C							
Property Progression at 21 °C	Symbol	Units	7 Days	14 Days	21 Days	28 Days	Test Standard
Glass Transition Temperature	T_g1_	°C	40	43	44	45	ISO 6721 (DMA) [33]
**Cured Resin Properties**							
**Properties**	**Symbol**	**Units**	**28 Days at 21 °C**		**16 h at 50 °C**		**Test Standard**
Glass Transition Temperature	T_g1_	°C	45		49		ISO 6721 (DMA)
Ultimate Glass Transition Temp.	U_Tg1_	°C	53		-		ISO 6721 (DMA)
Tensile Strength	σ_T_	MPa	36.3		42.4		ISO 527–2 [34]
Tensile Modulus	E_T_	GPa	1.9		2.0		ISO 527–2
Tensile Elongation	ε_T_	%	49.6		31.2		ISO 527–2
Flexural Strength	σ_F_	MPa	61.8		67.0		ISO 178 [35]
Flexural Modulus	E_F_	GPa	1.8		1.9		ISO 178
Flexural Elongation	ε_F_	%	>12.0		>12.0		ISO 178
28 Day Water Uptake	-	mg (%)	-		28.4 (0.6)		ISO 62 [36]
ILSS	X_ILSS_	MPa	-		32.3		ISO 14130 [37]

**Table 3:** Mechanical properties of the Ampro Bio resin composite reinforced with flax fabric determined experimentally in accordance with the ASTM D3039 standard [38].

	E_t_	ɳ	F	σ	ε_B_
	MPa		N	MPa	%
x	3513.944	0.235795	1429.164	44.342	4.476
s	222.4824	0.026671	17.9491	0.673086	0.373032
V	6.331416	11.31093	1.255916	1.517928	8.33265

**Table 4:** Test plan for each cutting tool.

Configuration Number	Rotation Speed [rpm]	Feed Speed [mm/min.]
1	1000	100
2	3000	100
3	5000	100
4	7000	100
5	9000	100
6	1000	300
7	3000	300
8	5000	300
9	7000	300
10	9000	300
11	1000	500
12	3000	500
13	5000	500
14	7000	500
15	9000	500
16	1000	700
17	3000	700
18	5000	700
19	7000	700
20	9000	700
21	1000	900
22	3000	900
23	5000	900
24	7000	900
25	9000	900

**Table 5:** The values of the roughness parameter Sa for selected samples depending on the type of milling cutter.

No.	n [rpm]	f [mm/min.]	Mapal SCM400	DIN 6527AN	Dolfa2-Al	Dolfa1-Al	Dolfa1S-Al
			Sa [µm]	Sa [µm]	Sa [µm]	Sa [µm]	Sa [µm]
1	1000	100		3.827	5.175	3.133	3.566
2	3000	100			8.116	6.625	10.975
3	5000	100			14.181		14.718
4	7000	100					
5	9000	100					
6	1000	300	29.946	5.221	5.321		5.054
7	3000	300			5.668		5.260
8	5000	300		4.760	12,920		4.082
9	7000	300		11.210		7.410	10.088
10	9000	300				9.662	9.898
11	1000	500	22.145		8.215		8.860
12	3000	500			5.382		6.544
13	5000	500			9.338		4.002
14	7000	500					
15	9000	500					
16	1000	700			7.794		10.379
17	3000	700	27.974	4.76	5.339	6.738	5.678
18	5000	700			5.569	4.763	4.117
19	7000	700				6.148	5.917
20	9000	700	31.735	10.2839		5.964	9.253
21	1000	900			7.038		13.000
22	3000	900			5.718		6.041
23	5000	900			3.913		3.396
24	7000	900					
25	9000	900					3.566

## Data Availability

The data presented in this study are available on request from the corresponding author (due to privacy).
